# Mapping the Genetics of Snoring: Insights into Family Patterns

**DOI:** 10.2174/0113892029378464260218054551

**Published:** 2026-04-23

**Authors:** Sunil Kumar Kadiri, Prashant Tiwari

**Affiliations:** 1 Department of Pharmacology, College of Pharmaceutical Sciences, Dayananda Sagar University, Harohalli, Bengaluru South, Karnataka- 562112, India

**Keywords:** Snoring, genetic determinants, quality of life, anatomical, genetic markers, lifestyle

## Abstract

Snoring is an everyday sleep phenomenon that impacts the overall health and quality of life. This review article describes the complex interplay of genetic and familial factors in snoring behavior. The discussion begins by reviewing the anatomical and physiological basis of snoring, with particular consideration of how genetic factors may affect the shaping of the upper airway. The article explores several studies proving that snoring can be inherited, exposing family case studies that reveal the patterns of recurrence. This review focuses on the detection of certain gene-related markers of snoring and the role of exogenous factors, such as obesity and habits of living, that may intensify genetic predisposition. The analysis illustrates the importance of assessing the genetic risk and the potential of genetic counseling in familial snoring. The prospective lines of investigation to further understand the genetic basis of snoring and the implications for tailored therapeutic approaches have been underlined. This contribution aims to clarify the genetic underpinnings of snoring and to facilitate further research into its hereditary potential by synthesising current findings for interested readers. Genetic research has shown that snoring is associated with heritable traits related to the structure of the airway, muscle tone, and the distribution of fat. Technologies such as next-generation sequencing and CRISPR enable the identification of genetic variants for targeted therapeutic intervention. Genetic-based treatments are more effective, and this has led to a paradigm change in sleep care with personalized approaches to snoring management.

## 1 INTRODUCTION

### 1.1 Snoring and Its Prevalence

Snoring is a very common disorder, with people of all ages and genders getting affected by it all around the world, from different ethnic groups [1]. It has been estimated that around 45% of adults snore occasionally and about 25% snore on a regular basis [1], which emphasizes the fact that this disorder affects a significant percentage of the population. The effect of snoring is the vibration of the soft tissues of the oropharynx and nasopharynx as air passes through the relaxed upper airway during sleep [2-5]. This narrowing can be caused by anatomy but also by sleep-induced relaxation of muscle tone [6]. There are multiple factors that can affect how likely someone is to snore and how badly they snore. Men, especially over 40, are more prone to snoring, but women can also snore, particularly after they have gone through menopause, where hormonal changes have an impact on the respiratory and muscle tone [7]. Aging also contributes to the increased collapsibility of the airway due to the loss of elasticity in throat muscles [8]. Obesity also plays a large role in the risk for obstructive sleep apnea, as fat deposits around the neck may compress the airway and make partial obstruction more likely to occur during sleep [9-12]. Nasal blockage caused by allergies or infections, and sleeping on one’s back, promotes airway obstruction by pulling the tongue and soft tissues backward. Snoring, often considered benign, may be a sign of more serious pathology, particularly OSA, a disease characterized by repetitive cessations of airflow during sleep [13]. Snoring is a common phenomenon produced by the vibration of the upper airway tissues, which can be caused by the turbulent airflow during sleep. OSA is characterized by repeated collapse of the upper airway during sleep, leading to oxygen desaturations and sleep fragmentation. Unlike the case of benign snoring, OSA is a disease that needs to be diagnosed and treated owing to its cardiovascular and metabolic influence.

### 1.2 Sleep Quality and Health Implications

The consequences of snoring on health and sleep quality are not limited to disturbing a partner’s rest. Although snoring is generally regarded as a nuisance, not only the person who snores, but also their partner and the person who is close to them can suffer potentially serious consequences and secondary effects, especially if snoring is present in its frequent or severe form. Snoring indicates a partial upper airway obstruction during sleep and may lead to sleep fragmentation and reduced sleep quality not only for the sufferer but also for their bed partner [14-17]. Somebody who snores may be subject to recurrent airway obstruction, hampering their ability to reach the deeper stages of sleep, which are most beneficial, including slow-wave and REM sleep. They are also important for cellular turnover, brain function, and systemic physiological homeostasis. As a result, the person may experience tiredness, impaired mood, reduced mental function, and a general reduction in daily activity [18]. The prolonged inability to sleep well, due to multiple wakings during the night or light sleeping, can lead to long-term consequences such as memory loss, difficulties with concentration, and mood swings. In addition, long-standing sleep fragmentation as a result of snoring may predispose to other disorders such as anxiety and depression, since sleep deprivation is thought to exacerbate mental difficulties [19]. If snoring is more than an infrequent occurrence, it can be a sign of OSA, a disorder that involves regular pauses in breathing while asleep [20-25]. OSA is a severe sleep disorder that typically involves loud and frequent snoring [26]. These pauses in breathing may cause oxygen levels in the blood to drop, which can place an extra burden on the heart. Sleep apnea can cause several health problems, such as high blood pressure, heart disease, stroke, and diabetes if not treated. Fluctuations in oxygen levels, known as oxygen desaturation, trigger the body's stress response and secretion of stress hormones like cortisol that further contribute to cardiovascular and metabolic dysfunction [27-29]. Those with untreated sleep apnea may also have an increased risk for arrhythmias, an irregular heartbeat [30].

## 2 METHODS

A literature search was conducted *via* systematic searches on PubMed, Scopus, Web of Science, and Google Scholar using a predefined list of terms related to the study topic. The inclusion criteria were limited to peer-reviewed articles, English language, and studies published within the last ten years to ensure applicability and quality. Irrelevant or duplicate articles were excluded from further analysis (Fig. **1**). Data extraction was based on PRISMA principles and limited to information on the methodological approaches and outcome measures used, and on the reporting of reliability to enhance transparency and trustworthiness of the synthesis's findings.

## 3 THE ROLE OF GENETICS IN SLEEP DISORDERS

Genetics plays a big part in the onset and manifestation of sleep disorders. Research shows that certain genetic polymorphisms may influence sleep behavior, be potential predisposing factors for the development of sleep disorders such as insomnia, narcolepsy, or sleep apnea, and/or impact the body's circadian rhythm [31-36]. Mutations in some genes, for example, can alter sleep-wake patterns, and inherited traits can also influence how an individual's body responds to external factors, such as exposure to light or stress [37].

### 3.1 Genetic Propensities to Snoring and OSA

Heredity plays a significant role in snoring and sleep apnoea. Studies suggest that these disorders tend to run in families. For instance, genetically determined anatomical features of the upper airway can predispose to snoring or sleep apnea [38]. A variety of genes are involved in the airway, including those that control, together with the anatomy of the airway, and those that affect the muscle tone of the airway. However, a few specific genes have been linked to a larger tongue, a narrower throat, or an airway that relaxes during sleep (an obstructed airway is a key component of snoring or apneic attacks seen in sleep apnea sufferers) [39]. For OSA, certain risk-increasing genetic variants have been identified in studies, for example, those involved in the control of muscle tone, the response to oxygen, and the distribution of fat around the neck [40]. People who have a family history of sleep apnea are more likely to have the condition themselves, even if they don't have other risk factors such as obesity [41]. It is also possible that genetic predispositions affect the way a person responds to environmental factors, such as weight gain, which can worsen snoring and sleep apnoea [42]. Understanding more about these genetic effects may provide useful information about individualized treatment options, such as targeted therapies or interventions customized to the genetic profile of a person.

### 3.2 Genetic-based Studies of Sleep Disturbances

Genetic factors contribute significantly to many sleep disorders, including insomnia, narcolepsy, Restless Leg Syndrome (RLS), and sleep apnea [43]. RLS, also known as Willis-Ekbom Disease, is a nervous system disorder characterized by an irresistible urge to move the legs, often accompanied by uncomfortable sensations such as tingling, aching, or crawling. They tend to worsen during periods of inactivity, particularly in the evening or at night, leading to significant sleep disruption. Specific genes have been associated with circadian rhythms, immune regulation, and dopamine regulation. For instance, narcolepsy has been linked to the HLA-DQB1 gene, and an alteration in the MEIS1 gene has been linked to RLS [44]. In addition, genetic influences on airway anatomy and muscle tone are important in sleep apnea. This information indicates that up to 80% of our sleep requirements and susceptibility to sleep disorders are influenced by genetics, results that might enable more personalized treatment options [45].

## 4 ANATOMICAL FACTORS INFLUENCING SNORING

### 4.1 Genetic Contributions to Airway Anatomy

Airway anatomy is affected by genetic influences and plays a role in the predisposition to snoring and Obstructive Sleep Apnoea (OSA). Some gene variants may affect the development of the upper airway, leading to an increased risk of obstruction during sleep [46-48]. For example, some genes that influence muscle tone, cartilage formation, and fat accumulation around the neck may be involved in airway patency. The studies show that the airway is more likely to be blocked in those who are genetically predisposed to having narrower airways or larger tongues, causing them to snore or have an apneic episode [49]. In addition, the regulation of connective tissue, which can be influenced by genetics, may also play a role in airway collapse during sleep. There is evidence that certain skeletal features, known to be genetically linked, can reduce airway diameter and therefore increase the risk of sleep apnoea [50-52]. Familial studies suggest that relatives of sleep apnoea patients are more likely to have similar problems [53]. Understanding the genetic determinants of airway anatomy will also be critical in the development of directed therapies and interventions, for instance, based on patient-specific reconstruction or surgical approaches to manage such structural abnormalities [54-57]. Imaging and measurement methods are necessary to understand the anatomical cause of snoring, with both advantages and disadvantages. MRI and CT provide high-resolution, 3D images of the upper airway, allowing precise quantification of soft-tissue volume, airway size, tongue size, and fat deposits. Cephalometry is a simple, two-dimensional technique for measuring craniofacial form. These techniques have also identified major anatomical risk factors for snoring, such as a narrowed pharyngeal airway and retrognathia. These approaches are limited by factors such as cost, availability, and variability with body position and sleep state. Imaging is usually performed with the patient awake and in the supine position, which may lead to errors in assessing airway dynamics during sleep. Furthermore, the heterogeneity across studies renders standardization challenging, thereby impeding reproducibility. The combination of imaging and genetic analyses enhances the understanding of the influence of inherited factors on snoring-related anatomy. Soft-tissue growth or skeletal development–related variants may also be associated with MRI-derived traits through genotype-phenotype association studies. Mediation analyses can test whether the association between genetic factors and snoring is mediated by anatomical factors. This convergence of disciplines enables going beyond correlational relationships and clarifying the biological pathways from genotype to phenotype, ultimately advancing personalized risk prediction and intervention for snoring-related conditions. Genetic alterations in the neuromusculature of the upper airway and in the propensity for inflammation affect predisposition to snoring (Fig. **2**). Craniofacial features and the quantity of soft tissue surrounding the airway are additional risk factors for obstruction. Modified respiratory drive and pharyngeal muscle responsiveness potentiate the development of airflow limitation. Advances in multi-omics technology, imaging methods, and biomechanical modeling are providing deeper insights into such interactions and open the way for the development of individualized therapies directed at the genetic and physiological determinants of snoring. Enhanced integration strengthens translational sleep medicine insights.

## 5 FAMILIAL PATTERNS OF SNORING

### 5.1 Familial Clusters with a High Occurrence of Snoring

Several case reports/family studies have delineated the genetic underpinnings of the high familial rates of snoring and sleep apnea [58]. These studies often reveal familial trends, suggesting that genetics plays a large role in the pathogenesis of snoring and associated sleep illnesses. An interesting case report includes a family in which multiple members over several generations suffered from severe snoring and OSA [59-61]. It was found that people with a family background of these problems had a significantly greater likelihood of both sleep apnea and snoring. By genetic testing, the investigators identified common features among these family members, including a larger tongue, a smaller airway, and certain skeletal characteristics, such as a recessed jaw. These structural abnormalities were inherited and led them to develop airflow obstructions during sleep that caused the snoring and apnea. A further family study of snoring was of a family in which both parents and four of the five sons snored. The researchers said the affected individuals had higher body mass index (BMI) and common anatomical features, including a constricted airway and excess fatty tissue around the neck. These bodily traits were, in all probability, magnified by genetic abnormalities that increase resistance to airflow during sleep. The researchers found a prevalence of snorers in the family, and concluded that the inheritance of the anatomical prerequisites (including general obesity or specific airway disposition) significantly contributed to the occurrence of snoring in the family. In both families, genetic testing and family histories revealed the importance of inherited anatomical characteristics in the development of snoring and sleep apnea. These data are consistent with a model where genetic factors, interacting with environmental factors such as obesity, are key determinants for these sleep disorders. This may indicate potential strategies for customized treatment and prevention for families in which snoring is prevalent.

### 5.2 Analysis of the Heritability of Snoring Behaviors

Heritable analysis of snoring behavior is designed to investigate the extent to which genetic factors contribute to a predisposition to snoring. Heritability is the percentage of variance in a trait (*e.g*., snoring) attributable to genetic rather than environmental factors. Genetic studies on snoring analyze families or twins to determine the influence of genetic factors on snoring and co-existing sleep disorders [63]. Studies show that genetics plays a major role in snoring. For instance, twin studies show that snoring is more common in monozygotic twins who share virtually all their genes than in dizygotic twins who share approximately half [64]. This indicates a genetic predisposition to snoring. In addition, family studies show that snorers’ relatives are more likely to snore, and snoring is familial. These findings suggest that inherited features, such as airway dimensions, muscle tone, and fat distribution around the neck, may determine whether a person is likely to snore [65-68]. Genetic studies identified certain genes related to airway size, muscle function, and fat metabolism that might increase the likelihood of snoring for some [69, 70]. However, snoring is also heavily influenced by environmental factors such as obesity, smoking, alcohol intake, and sleeping position, all of which may interact with genetic factors and exacerbate the condition in a joint genetic-environmental manner [71].

## 6 GENETIC MARKERS RELATED TO SNORING

Studies focused on genetic markers related to snoring are still in their early phases; several reports have identified different gene candidates that may make certain people at risk. Snoring occurs when tissues in the throat vibrate as air flows past them, because the airway is partially closed during sleep. Usually, these genetic markers relate to anatomical features, muscle tone, fat distribution, and airway structure that influence snoring [72].

### 6.1 The Hunt for Snoring-specific Genes

The search for snoring-specific genes is an active area of investigation. Although the involved genes are not yet known, several studies indicate that some genes may influence snoring by affecting airway anatomy, muscle tone, and fat distribution [73].

Here are a few genes and genetic influences related to snoring:

#### 6.1.1 Genes Associated with Craniofacial Morphogenesis

FGFR2 (Fibroblast Growth Factor Receptor 2): This gene is key to craniofacial morphogenesis and may influence the size and shape of the jaw, palate, and upper airway [74-77]. FGFR2 mutations have also been associated with traits that might increase risk for snoring, *e.g*., a smaller airway or retruded jaw [78]. COL1A1 and COL5A1 (Collagen Genes): These genes play an important role in collagen production, which is a key component of connective tissues. Mutations or variants in these genes may also weaken the tissues surrounding the airways, potentially resulting in a heightened probability of snoring or collapsing airway walls [79] (Table **1**). Recent animal studies based on CRISPR have demonstrated the potential of snoring-related gene targeting to improve upper airway patency. One important example is the FGFR2 editing, which is key for craniofacial development and airway formation. In murine models, CRISPR-Cas9 was also used to correct gain-of-function mutations in FGFR2, associated with craniosynostosis syndromes often accompanied by airway restriction and snoring. The gene editing stimulated the growth of the midface and nasal airways, which resulted in decreased airway resistance. These therapeutics exhibited a high level of target specificity, having low off-target effects as confirmed by good manufacturing practice and off-target prediction tools. The delivery method, usually using AAV (adeno-associated virus) vectors tailored for respiratory tissues, made delivery of CRISPR components more precise and localized. Other genes linked to the shape of the airway itself, including PAX9 and TBX22, have been tested in similar preclinical models with promising outcomes aside from FGFR2. These studies demonstrate the ability of gene editing to correct anatomical contributors to snoring and sleep-disordered breathing. There remain roadblocks to clinical translation, including delivery efficiency and long-term safety, but the precise targeting demonstrated in these animals paves the way for the development of therapeutic strategies for congenital or developmental airway dysfunction associated with snoring.

#### 6.1.2 Genes Regulating Fat Metabolism and Distribution

ADIPOQ (Adiponectin): This gene controls fat metabolism and is involved in the distribution of fat in the body [83]. Too much fat building up in the neck can press on the airway and become a major contributor to snoring. ADIPOQ gene polymorphisms may influence the susceptibility to the accumulation of neck fat that is enough to cause snoring and obstructive sleep apnea. Collagen-, fat-, and muscle-related genes show age-dependent expression profiles, which are governed by intrinsic genetic programming as well as by epigenetic alterations. Reduced collagen synthesis with age results in decreased skin elasticity and structural integrity. Altered gene expression involves changes in regional fat distribution, ultimately leading to metabolic perturbations and increased adiposity. Genes related to muscle tone, in particular those involved in protein synthesis and neuromuscular function, become downregulated, and sarcopenia ensues. Epigenetic processes, including DNA methylation and histone modifications, influence gene expression and are responsive to environmental exposures, lifestyle, and hormonal changes associated with aging, and are determinants of tissue integrity and functional capacity over the course of an individual’s life. Epigenetic modifications, primarily DNA methylation, are essential for the regulation of gene expression without altering the underlying DNA sequence. In the aging process, these changes influence ARMs - airway muscle tone, inflammation, and circadian rhythms, which are the key biological elements involved in snoring and sleep-disordered breathing. Age-related changes in the methylation of genes like FOXO3, TNF-α, and CLOCK – involved in oxidative stress response, inflammation, and sleep regulation – may thus influence different snoring phenotypes (such as chronic snoring and OSA-associated snoring). It has recently been shown that some individuals with particular characteristics of snoring have specific epigenetic signatures that could potentially be used as biomarkers of disease severity or progression. Genes involved in muscle tone control may be hypermethylated, so the upper airway is less stable during sleep, increasing the risk of vibratory snoring in the elderly. Hypermethylation of the COL1A1 promoter is associated with reduced gene expression, which leads to decreased production of collagen and, consequently, to weakening of the airway wall [84]. Similarly, epigenetic silencing of anti-inflammatory pathways could promote snoring severity by enhancing airway inflammation. Understanding these age-related epigenetic changes provides a mechanistic link between genetic predisposition, environmental factors (*e.g*., smoking, obesity), and the phenotypic manifestation of snoring. This knowledge provides opportunities for the development of personalized medicine or preventive approaches targeting epigenetic changes to reduce snoring and its related health burden.

LEP (Leptin): A hormone involved in regulating body weight and fat storage. Studies show that certain variants of the LEP gene may be associated with obesity and neck fat deposition, a major risk factor for snoring [85, 86].

#### 6.1.3 Genes Controlling Muscle Tone

*ACTN3 (Alpha-Actinin-3):* This gene encodes an actin-binding protein with a structural function in skeletal muscle. ACTN3 polymorphisms have been linked to the ability of muscles to generate force and to their endurance, which would contribute to the tone of the muscles surrounding the airway during sleep [87].

Although it is well known that genetic variants also influence the risk of snoring, recent studies have also begun to underline that snoring is not a simple behavioural problem but is influenced by genetics. Several genes that predispose to physical characteristics associated with snoring have been identified in several studies [88]. Prominent was the ADRB2 gene, encoding the beta-2 adrenergic receptor, which is intimately involved in the regulation of airway smooth muscle (Fig. **3**). Allelic variants in this gene have been suggested to alter the relaxation of upper airway muscles during sleep and to predispose to airway collapse, a major cause of snoring [89]. Furthermore, variations of the tissue remodelling gene MMP9 have been linked with changes within the upper airway that may predispose a person to snoring [90-92]. Studies show that these genetic influences determine the airway's physical nature, which the airway is more likely to collapse or vibrate (causing snoring) during sleep. Results: What is especially intriguing is the identification of genes involved in sleep, including the circadian rhythm regulator CLOCK [93]. Variants in these genes could influence the sleep architecture, particularly sleep stages associated with muscle relaxation in the airway, which may result in snoring. In addition, a genetic component within families has been suggested [94], with snoring possibly being familial through common genetic components. Twin studies have further confirmed this, indicating that monozygotic twins have a greater concordance rate for snoring than dizygotic twins. However, although genetic variants are important, environmental and lifestyle factors (including obesity, alcohol consumption, and sleeping position) are also important contributing factors to snoring. Further studies are needed to identify the full spectrum of genetic influences and the way they interact with these factors.

#### 6.1.4 Genes Implicated in Snoring or Sleep Apnea in Genome-wide Association Studies (GWAS)

Several loci have been identified that are related to snoring and OSA , and both disorders show moderate-to-high heritability. Major results include polymorphisms in genes nearest to or within craniofacial, respiratory, and obesity-related genes. The FTO (fat mass and obesity-associated) region is also associated with obesity, which is a major risk factor for sleep apnoea. FTO polymorphisms might have an effect on obstructive sleep apnea *via* influencing body mass and the fat localization. Other obesity-related genes, such as GPR75 and LEPR, have also been linked. Genomic regions close to TBX5, a gene known to play a role in upper airway and thoracic organ development, have been associated with snoring. Inflammation-related genes, CRP (C-reactive protein) and IL6, have also shown associations suggesting inflammatory mechanisms may influence collapsibility of the airway or respiratory control. In addition, loci were identified near the circadian rhythm genes ARNTL and PER1, associated with sleep timing and respiratory symptoms, respectively. These findings highlight the polygenic and multifactorial nature of snoring and OSA, in which genetic influences encompass anatomical, metabolic, and neurological pathways. Further research is underway to clarify causal variations and pathways.

### 6.2 Genetic Predisposition to Snoring: Systems Biology Perspective

Snoring is a genetically predisposed, physiological, and environmental-related disease. A systems biology view, based on the integration of genomic, transcriptomic, and epigenetic information, presents a comprehensive model for understanding genetic background heterogeneity in snoring. This approach analyses how anatomical structures, neuronal control, and metabolic processes interact, and it is applied in the field of the genetic basis of snoring [95] (Table **2**). Genetic predisposition plays a large role in influencing craniofacial shape, upper airway muscle tone, and breathing mechanics. Genetic changes affecting the soft palate, tongue, and tissues of the pharynx may cause airway obstruction during sleep that results in snoring. In addition, mutations in genes regulating muscle function affect airway muscle relaxation, exacerbating soft-tissue vibration and airway constriction. These genetic contributions to snoring are also influenced by obesity, as excess adipose tissue around the neck makes it harder for air to flow. Systems biology perspective includes the neurogenetic control of breathing as well as anatomical features. Neuronal circuits underlying the regulation of respiratory patterning and airway responsiveness are modulated by genes that influence the severity of snoring. Genetic differences in multiple neurotransmitter receptors and signaling cascades may alter respiratory control, compromising airflow regulation and predisposing individuals to snoring. Epigenetic modifications such as DNA methylation and histone acetylation modify gene expression and thus may contribute to the snoring phenotype. Computational and machine learning–based genomic analyses empower the identification of genetics markers for snoring in studies. By integrating large-scale data sets from Genome-Wide Association Studies (GWAS), transcriptomics, and proteomics, predictive models of patients predisposed to snoring and related disorders such as OSA can be constructed. This finding allows for individualized care and targeted intervention based on genetic risk profile. In addition to this, gene-environment interactions matter, such as postural sleeping, dietary, and lifestyle habits. Systems biology studies these multi-layered interactions and recognizes that genetic predisposition is not a sole determinant of snoring severity. An individual's snoring patterns are influenced by a complex interplay between genetic susceptibility and external factors. Systems biology provides a holistic perspective on the genetic foundation of snoring by compiling evidence from multiple layers of biology. This integrated concept enhances our understanding of airway physiology and contributes to the development of preventive and decelerative measures to minimize snoring and improve sleep quality. Ultimately, the application of genetics within systems biology is driving sleep medicine forward, with the promise of leading to more effective and personalized approaches to respiratory health.

## 7 ENVIRONMENTAL AND LIFESTYLE INTERACTIONS

Snoring is modifiable by environmental and lifestyle factors of the snorer and bed partner. Airflow through the nose can be influenced by environmental factors such as air quality, allergens, and room temperature, which may be associated with increased snoring [96]. Moreover, certain habits like obesity, alcohol consumption, smoking, and sleeping on your back can worsen snoring by relaxing the muscles of the throat or increasing the resistance of the airways. Reduced physical activity and poor sleep hygiene may additionally exacerbate these problems. By treating these issues — losing weight, quitting smoking, improving sleep patterns, and avoiding environmental triggers — people can significantly eliminate, if not prevent, snoring [97].

### 7.1 The Interplay of Genetics and Environment

The interaction between genetics and environmental contributors, including obesity and smoking, is informative of snoring. Some people have inherited genes that make them more likely to have anatomical features that contribute to snoring, such as a smaller airway, larger tonsils, or weaker throat muscles [98]. These properties can cause airway obstructions during rest, causing snoring. Yet not all instances of snoring can be traced back to genetics; environmental factors are the key culprit in making snoring worse [99]. For example, being overweight is an important environmental risk factor. Being overweight, particularly in the neck, can also raise the risk of airway obstruction as a result of fatty deposits [100]. This added pressure compresses the upper airway, forcing it to vibrate and produce snoring sounds. Smoking exacerbates this risk, as it agitates and inflames the lining of the nose and throat, resulting in more resistance to the airway. Toxic components in cigarette smoke can lead to chronic inflammation and swelling, which exacerbates snoring. In addition, smoking may worsen conditions such as sleep apnea, which is associated with loud and frequent snoring. The interaction of physical and environmental factors with genetics leads to a cycle in which genetics lead to greater susceptibility, and physical and environmental influences exacerbate the condition, making management more difficult [101]. Both are important, inherited and lifestyle factors, if you want to get rid of snoring and improve your respiratory health effectively. The gene-environment approach to snoring has several drawbacks. The complexity of the link between genetic inheritance and environmental factors challenges embracing specific contributors. The predictive value of genetic testing may be overstated, since alterable factors such as obesity and alcohol intake have a more powerful impact. In addition, the cost of genetic testing may not justify its routine use due to the low clinical yield. Although genetic predisposition has been identified, lifestyle measures continue to be the primary mode of treatment. In conclusion, evidence of gene–environment interactions related to snoring is still lacking, and further investigation is needed to improve prediction models and develop targeted and cost-effective interventions. Gene-environment (GxE) analyses of snoring are based on various methods and study designs to investigate the interplay between genetic predispositions and environmental exposures such as body mass index (BMI), smoking, alcohol consumption, and sleep behaviors. One common approach is to conduct stratified genome-wide association studies (GWAS) that perform analyses within environmental strata (*e.g*., smokers *vs*. non-smokers) to detect heterogeneous genetic effects. (interaction terms, *e.g*., genotype × BMI) investigated by linear or logistic regression-based interaction models to determine whether environmental factors modify genetic associations with snoring. To achieve greater precision, these researches often combine polygenic risk scores (PRS) with information on environmental exposures to predict the risk of snoring. Additional methods, inclusive of multivariate regression, mixed models, and Mendelian randomization, may provide further improvements in assessing causality and addressing reverse causation. It is important to control for confounding factors. Variables like age, sex, ancestry, socioeconomic status, and comorbidities such as sleep apnea or obesity are typically adjusted for to ensure genuine gene-environment interactions. Population stratification is accounted for with principal component analysis or mixed models, while repeated measures or longitudinal data improve temporal inferences. These approaches enable the investigation of complex interactions, thus informing the pathways through which lifestyle and environmental factors alter genetic risks for snoring and improve risk prediction and intervention. The statistical and mechanistic understanding of gene-environment interactions for snoring in the future looks to be enhanced by the integration of GWAS with studies of environmental exposures. Advanced approaches such as polygenic risk scores and Mendelian randomization may further strengthen causal interpretation. Moreover, the combination of longitudinal cohort studies allows for dynamic assessment of genetic predisposition and lifestyle-related factors such as obesity, alcohol consumption, and airway inflammation. Multi-omics approaches, including epigenomics and transcriptomics, provide additional insights into the biology of snoring vulnerability. Improving statistical models through machine learning and Bayesian methods will yield better predictions and a deeper understanding of gene-environment interactions in sleep-disordered breathing (Table **3**).

### 7.2 Age Effect on Snoring

Age is an important factor in snoring, as changes in physiology over time can lead to airway closure and vibration during sleep. The tone of the throat and tongue muscles usually weakens with age, and the tissues are more likely to collapse and block the airways. This result is an increase in snoring, in both its quantity and quality. Aging is associated with decreased airway tissue elasticity, which may further contribute to airway narrowing and vibration.

Weight gain is commonly related to the aging process. Excess fat in the neck region may compress the airway, disrupt airflow, and lead to collapse during sleep. The elderly are also more likely to suffer from conditions such as sleep apnea, which can increase snoring as a result of repeated airway obstructions. Ageing can cause changes to sleeping patterns. Deepest sleep stages might decrease in number to yield more snoring from lighter sleep, or more intense snoring. Hormonal changes, mainly in postmenopausal women, might influence the tonus of the muscles of the airways and the severity of snoring. Snoring is common in aging and can be prevented or at least minimized through a healthy lifestyle with regular physical activity, weight control, and an adequate sleep routine. Persistent snoring should be evaluated, especially if it disrupts sleep or indicates a potential health issue. With ageing, lung fibroblasts undergo a dramatic reduction in COL1A1 (type I collagen alpha-1 chain) transcription and thereby produce less collagen I in the airway extracellular matrix. Collagen I is necessary to maintain tensile strength and elastic properties, combined with elastin. At the same time, aging promotes the accumulation of advanced glycation end-products (AGEs), including glucosepane, that cause non-enzymatic cross-linking of collagen fibers. The cross-links increase the stiffness of the matrix and reduce its elasticity by limiting the physiological sliding and stretching of the collagen fibrils. The synergistic impact of diminished collagen production (through decreased COL1A1 expression) and the stiffening of the residual matrix due to AGE cross-linking compromises airway wall elasticity. The diminished elastic recoil heightens airway collapsibility and resistance, promoting turbulent airflow during sleep, which consequently results in snoring.

### 7.3 The role of sleep position and habits

Sleep position and habits significantly influence how often and how severely a person snores. When someone sleeps on their back, it can worsen snoring because gravity pulls the tongue and soft tissues in the throat backward, which partially blocks the airway and causes vibrations that result in snoring sounds [105]. On the other hand, side sleeping may relieve this problem because the airway remains more open. There are six proven-based approaches to stop snoring and improve sleep quality through the application of certain lifestyle changes and behavioral modifications. Controlling one's weight decreases the airway obstruction, and changing the sleeping position (avoid supine position) reduces the airway collapse. Limiting alcohol and sedatives reduces the relaxation of the throat muscles and thus helps decrease the severity of snoring. Promoting good sleep hygiene may increase airway muscle tone and improve overall sleep stability. Improved efficiency of breathing may be brought about by improving nasal airflow using decongestants or nasal strips. Doing oral and breathing exercises like myofunctional therapy can increase the airway muscle strength. These treatments offer a non-invasive, evidence-based approach to stop snoring and restore refreshing sleep (Fig. **4**). The muscles, including those in the throat, relax more when a person is sleep-deprived; this can further increase airway resistance and lead to worsening snoring [106]. A regular sleep schedule, good sleep posture, and enough sleep help reduce the frequency and severity of snoring because they tend to promote better function of the airway and less muscle relaxation while a person sleeps [107].

### 7.4 Genetic and Lifestyle Interactions in Snoring Susceptibility: Heritability Estimates and Quantitative Associations

The genetic background plays an important role in snoring predisposition, and the proportion of variation explained by genetics is between 10 and 40% [108]. Several snoring-associated loci have been identified by GWAS (most notably DLEU7 and MSRB3) and are related to the structure of airways and neuromuscular control. Moreover, results from polygenic risk scores analyses are consistent with those individuals with high genetic risk for obesity and craniofacial morphology being more likely to be habitual snorers. Lifestyle factors such as Body Mass Index (BMI), drinking, and smoking showed significant associations with snoring. Mendelian randomization indicates a causal association between increased BMI and snoring, and this association in obese individuals is around 1.5 for the Odds Ratio (OR). Smoking is related to increased airway inflammation and heightened risk of snoring, while consumption of alcohol leads to relaxation of muscles in the airway and potential worsening of obstruction. Together, this genetic-lifestyle interplay indicates a complex nature of snoring as a sleep trait. Genetic factors determine baseline risk, and modifiable behaviors have a substantial impact on the degree of snoring. Specific interventions focused on weight loss and smoking cessation may mitigate genetic risk and, therefore, represent a tailored approach to reducing snoring prevalence.

#### 7.4.1 Methodological Limitations

A major methodological barrier to studies employing Polygenic Risk Scores (PRS) and Gene-Environment (G×E) interaction models is the inherent assumptions and limitations that impact prediction accuracy and causal inference. However, polygenic risk scores based on currently identified variants explain only a small portion of phenotypic variance, particularly in diverse populations with different patterns of linkage disequilibrium and allele frequencies. This curtails their predictive power and poses challenges regarding portability and fairness. In addition, G×E models, in particular those applying Mendelian Randomization (MR) to establish causality, rely on strong assumptions: genetic instruments need to be strongly associated with the exposure, influence the outcome only *via* the exposure (exclusion restriction), and should not be confounded by any other variables. Violations of these assumptions, including horizontal pleiotropy or population stratification, can bias the estimates and lead to spurious causal inference. Moreover, MR often assumes that genetic and environmental factors operate additively and without gene-environment interactions, thereby oversimplifying complex biological pathways. These limitations underscore the need for more comprehensive sensitivity analyses, improved genetic instruments, and diverse population data sets to enhance inference and ensure generalizability. In the end, PRS and MR remain powerful tools, but overinterpretation of their methodological challenges should be avoided to foster robust applications in genetic epidemiology.

## 8 ASSESSING GENETIC RISKS IN SNORING

Evaluation of genetic risks in snoring is derived by looking at hereditary elements that affect the airway anatomy, muscle tone, and lung function [109, 110]. Genetic testing and analysis may assist in determining these predispositions, offering the possibility of early intervention and tailored treatment approaches [111]. People who have close relatives who snore or have sleep apnoea may have an increased genetic risk. However, it is not just genetics that determines whether someone is a snorer: environmental factors, including obesity and smoking, contribute significantly to its onset.

### 8.1 Comprehensive Approaches to Genetic Risk Assessment: Instruments, Counseling, and Family History Reviews

Among the instruments to assess for genetic risk in the individual are genetic testing, family history analyses, and high-resolution genomic profiling. Genetic testing analyzes a person's DNA to detect specific genetic changes associated with conditions that may increase a person's risk of snoring or sleep-related disorders [112]. For example, some genetic polymorphisms can be associated with a greater prevalence of anatomical features that underlie snoring, such as a narrow airway, large tonsils, or reduced throat muscles. Family history reviews are also important in assessing genetic risk. Patterns of snoring, sleep apnea, or other sleep disorders tend to run in families, and by identifying them within families, doctors can determine whether a genetic link exists. This also helps determine who should be targeted for interventions to reduce their risk of snoring due to family history [113]. Additionally, high-resolution genomic profiling, a more advanced instrument, entails sequencing a person's whole genome to detect myriad genetic variants potentially related to sleep issues. Such comprehensive analyses (10) clearly demonstrate specific gene-environment interactions that contribute to snoring and other sleep disorders. When used in conjunction with clinical assessments, these genetic tools enable clinicians to offer individuals a more personalised snoring management strategy, resulting in early intervention and/or specific treatment options based on a person's unique genetic composition [114]. Genetic counseling and family history evaluation are important tools for studying hereditary traits related to snoring. Genetic counseling can help people understand how genetics may be involved in snoring, particularly if there is a history of sleep disorders or conditions such as sleep apnea in the family (Fig. **5**). Counselors help individuals understand the influence genetic predispositions may have on the structure of the airway, muscle tone, and breathing. Ancestral evaluations are beneficial for identifying instances of snoring or sleep-related problems across generations of a family, suggesting the presence of hereditary factors [115]. This may be very useful for early interventions, lifestyle modifications, or other specific therapies to control or reduce the risks associated with snoring [116-118]. Analyses stratified by sex and age may be conducted if there is sufficient statistical power, noting that the heterogeneity in snoring phenotypes is well-established. Different hormonal patterns, airway differences, fat deposition, gender, and age-related physiological changes might affect the patterns of snoring. Stratification allows for a better understanding of these differences and may identify subgroup-specific associations or risk factors. By analyzing potential effect modifiers and enabling more tailored prevention and intervention strategies for different populations at risk of snoring, this strategy aims to increase the relevance and interpretability of the results.

### 8.2 Possible Misuses of Direct-to-consumer Genetic Testing (DTC-GT)

Direct-to-Consumer Genetic Testing (DTC-GT) allows a person to obtain genetic information without the involvement of medical professionals; however, concerns arise in its use for assessing the risk of snoring. Several DTC-GT companies advertise their capacity to detect genetic variants associated with behaviors (including snoring) by referencing SNPs in genes such as FGFR2, COL1A1, or in areas associated with obesity. However, these relationships are usually polygenic, weak, and influenced by the environment and anatomy. Without professional guidance, people might mistakenly interpret probabilistic outcomes as being deterministic, which could lead to unnecessary anxiety, false comfort, or the utilization of unproven interventions. An individual could also incorrectly self-diagnose obstructive sleep apnea because of a genetic mutation for “snoring risk” and therefore delay clinical evaluation. On the other hand, people without risk factors may choose to ignore real symptoms. In addition, the companies are often not subject to government regulation, and the reports can contain errors, be unsubstantiated, or be misleading. As such, while DTC-GT has educational value, overinterpretation of snoring risk may cause mental stress, delay diagnosis, or lead to inappropriate use of healthcare resources without professional guidance.

## 9 FUTURE RESEARCH DIRECTIONS

### 9.1 New Genetic Research Tools and Methods

Recent advances in genetics are rapidly making it clear that there are many causes of snoring. Although snoring was previously considered a mild nuisance, emerging evidence suggests that heredity plays a major role in the condition and is often linked to associated conditions such as obstructive sleep apnea. A major instrument in this study is Next-Generation Sequencing (NGS), which allows researchers to analyze the entire genome of people with snoring problems [119]. By analyzing the DNA from both snorers and non-snorers, researchers are also identifying particular genes that may be associated with anatomical features such as airway size and shape, muscle tone, the amount of fat around the neck, and perhaps even the way we breathe while we sleep. The CRISPR-Cas9 gene-editing technique appears to be very promising as well, enabling researchers to modify genes associated with snoring and study them in animal models [120]. CRISPR-Cas9 gene editing technology provides a new approach to probe for potentially treating snoring by targeting genetic factors related to airway structure, muscle tone, and respiratory control. This refined genetic engineering technique enables scientists to alter specific genes linked to snoring and assess their physiological effects in well-defined animal models. With these alterations in genetic sequences, researchers can study molecular processes that contribute to snoring, leading to a cause-and-effect understanding of how certain genetic variants affect snoring and airway blockage. These studies enable personalized treatments by offering guidance to explore directed drugs that have the potential to target the genetic cause of snoring as opposed to its symptomatic relief. In addition, the applications of CRISPR extend beyond research, as refined gene-editing techniques may eventually offer individualized treatment options to those at risk for snoring and related sleep disorders such as obstructive sleep apnea. Although ethical and regulatory challenges persist, CRISPR-Cas9 has the potential to revolutionize sleep health in the near future through precision medicine and genetic therapeutics. In addition, the marginal benefits of machine learning and bioinformatics are enabling the analysis of large genomic datasets, making it increasingly possible to accurately identify genetic variants and their potential relationships to snoring. These advances are paving the way for personalized treatments, in which genetic information may be used to develop individualized therapies, like a drug or potentially genetic modifications to target and even remove snoring. As this line of inquiry advances, these breakthroughs could bring about more efficient, customized treatments for a large portion of the population suffering from this widespread, yet undeniably disturbing concern.

Considerable progress has been made with CRISPR/Cas technology in the context of genetic disorders. Major findings and advances are as follows:

A review highlights the growing number of clinical trials utilizing CRISPR/Cas for gene therapy, addressing modes of action, delivery systems, and latest developments [121].

An Approved CRISPR Therapy: The first-ever CRISPR-based drug, Casgevy, was approved for treating sickle cell disease and beta thalassemia. This treatment, which works by stimulating the BCL11A gene involved in producing fetal hemoglobin, represents a curative, one-time therapy [122].

Broadening Disease Targets: CRISPR is also being studied as a treatment for a range of diseases, including cancer, AIDS, cystic fibrosis, muscular dystrophy, Huntington’s disease, and COVID-19. Recent clinical trials: The field is rapidly evolving, with new studies focused on autoimmune disorders and different genetic conditions.

### 9.2 Opportunities for Individualized Treatments for Snoring and Sleep Health

Advances in genetics, technology, and data science now support personalized approaches to treating snoring, rather than older, common approaches. Genetic sequencing has identified variants that influence airway shape and size, muscle tone, and fat disposition, all of which contribute to snoring. The use of these insights in conjunction with real-time sleep monitoring (including wearable devices and home studies) enhances personalized treatment decisions. The applicability to the clinical setting of the genetic findings is limited, as GWAS show minimal effects and Polygenic Risk Scores (PRS) possess limited predictive potential. Combining Polygenic Risk Scores (PRS) with clinical interventions may thus represent a promising strategy for gene-guided treatment stratification for snoring. PRS might also predict individuals with a higher genetic predisposition for related conditions based on craniofacial morphology, neuromuscular tone, or airway collapsibility. Those identified as having an increased PRS may be sent for structural interventions, such as maxillomandibular advancement or nasal surgery, while those with a reduced genetic risk may be candidates for CPAP or behavioral treatments. This approach enables more precise, individualized care by linking genetic signatures to treatment responses. In the end, PRS-based clinical decision-making enhances translational relevance, enabling early, targeted intervention and, subsequently, better outcomes in patients with sleep-disordered breathing. Tailored interventions face challenges in translating genetic knowledge into practice, largely due to the difficulty of incorporating multi-omic data (*e.g*., genomics, imaging, and environmental data) into actionable models of care. Current treatments for OSA, including CPAP, oral appliances, and lifestyle adjustments, do not take into account genetic subtypes, and no tailored treatments for the genetic routes of snoring are currently available [123, 124]. The processing of genetic data is complicated by ethical issues, including privacy, ownership, and the potential for discrimination in insurance or employment. Informed consent should also address these risks in light of the final cardiovascular implications of snoring , which often are not considered. In the case of direct-to-consumer genetic testing, the lack of stringent regulation represents a serious problem, as it can lead to misunderstanding of the data and commercial exploitation. Precision medicine holds promise for the treatment of snoring; however, resolving technical, ethical, and clinical issues is mandatory before its routine clinical application. A thorough approach is required to promote clinical and ethical debate on the applicability of genetics in snoring and the mechanisms involved. From a clinical perspective, identifying genetic markers predicting airway obstruction and neuromuscular control may enable improved diagnosis and customized therapeutic strategies, potentially including targeted PGx. Ethical issues are concerned with genetic privacy and security, informed consent, and the potential for stigmatization in predictive testing. The responsible use of genetic information in this context requires balancing medical advancement with individual patient autonomy and access to care. Additionally, insurance and employment discrimination must be precluded. Fostering productive cross-disciplinary interactions among geneticists, clinicians, and ethicists will be critical to the ethical and fruitful translation of future genomic discoveries into sleep medicine.

#### 9.2.1 Ethical Considerations in Gene Therapy

Somatic (non-heritable) genome editing, which takes place in an individual's cells, is being used in therapy for diseases like HIV and sickle-cell disease. The WHO supports its application under appropriate control within specified clinical and ethical conditions, including safety monitoring, participation, registries, and international governance. Meanwhile, germline (heritable) editing that involves altering DNA in embryos or gametes and passing changes downstream to future generations is currently viewed as irresponsible by the WHO and raises moral and safety concerns [125]. Clinical uses of human germline editing have been unequivocally reaffirmed by the WHO RIS as not appropriate to proceed at this time. The key differences are a degree of danger and a degree of ethical oversight: Somatic alterations affect only the treated individual, and are well within the regulatory regimes already in place; changes to the germline, on the other hand, involve unknown potential multi-generational risks, and are the subject of international bans, registries, and appeals for caution and transparency [126].

## CONCLUSION

Recent advances in genetics have increased our understanding of hereditary snoring, with a complex interplay between airway anatomy, neuromuscular control, and fat distribution. GWAS have also identified a number of genetic variants shared with snoring, reinforcing a pattern of familial inheritance. Snoring is a complex trait influenced by genetics, environment, and epigenetics. NGS and CRISPR-Cas9 enable precise detection of genetic markers associated with airway obstruction, respiratory mechanics, and neuromuscular control. Using these and other tools, scientists can probe cause-and-effect relationships between genetic variations and the physiological processes that contribute to snoring, ultimately leading to novel, personalized therapeutic approaches. The incorporation of genomic information with sleep-tracking devices, AI-powered predictive models, and multi-omic data results in improved treatment design, moving generalized interventions toward focused therapies. Personalized medicine may lead to improved levels of care for snoring *via* tailored interventions (surgical correction, neuromodulatory therapy, and airway remodeling) based on genetic factors. Data privacy, informed consent, and equitable access to genetic-based therapies are among the ethical issues that must be addressed to promote responsible clinical translation. The integration of genetic knowledge into sleep health approaches has the potential to improve diagnostic capabilities, increase treatment efficacy, and mitigate long-term health consequences strongly associated with chronic snoring and sleep-disordered breathing.

## Figures and Tables

**Fig. (1):**
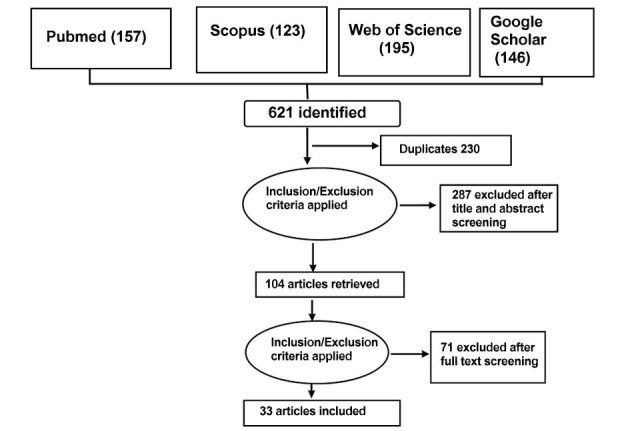
PRISMA flow diagram.

**Fig. (2):**
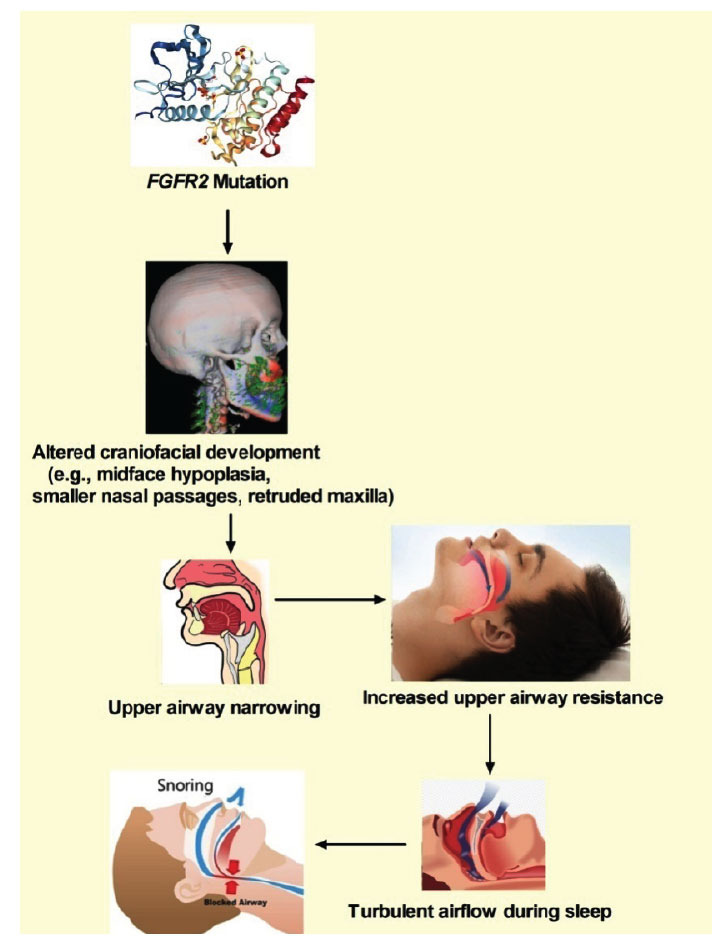
Gene-anatomy-physiology causal pathway diagram involved in snoring. The craniofacial anatomical illustration in this figure is reproduced from [Wollny *et al*., BMC Medical Imaging, 2008] under the Creative Commons CC BY license [48].

**Fig. (3):**
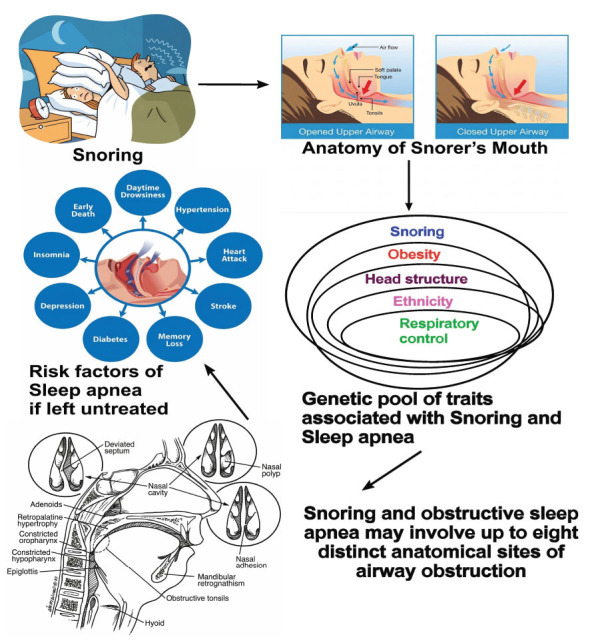
Genetic pool of traits and anatomical influences of snoring: Pathophysiological links to sleep apnea.

**Fig. (4):**
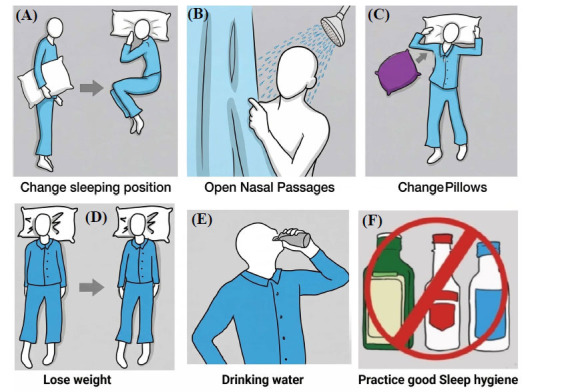
Six evidence-based strategies (A to F) for reducing snoring and enhancing sleep quality.

**Fig. (5):**
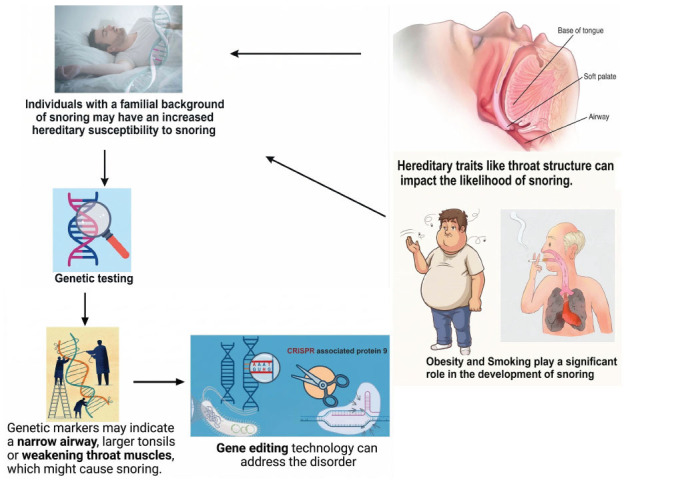
Evaluating genetic influences and family medical history in relation to the heritability of snoring.

**Table 1:** Role of *COL1A1* variants in airway collapse.

**Category**	**Details**
Gene	*COL1A1* (Collagen Type I Alpha 1 Chain)
Gene Function	Type I collagen is produced, which is essential for the structural integrity of connective tissues, including the walls of the airways.
Role in Airway Structure	Imparts mechanical strength and stiffness to tracheal and pharyngeal tissues.
Effect of Variants	Missense or regulatory variants may diminish collagen quality or quantity, thereby compromising airway support.
Associated Phenotypes	Tracheomalacia
	Narrowed or floppy airways
	Increased susceptibility to airway collapse during sleep
Evidence from Studies	Chen Z *et al.* (2024) : GWAS associated COL1A1 SNPs with snoring and airway diameter [80]
	He H *et al.* (2021): COL1A1 mutant mice exhibited airway softening [81]
	MarincakVrankova Z*et al.*(2023): A human cohort demonstrated the association of COL1A1 polymorphisms with obstructive sleep apnea. [82]
Clinical Implications	May function as a genetic marker for airway collapsibility, contributing to the prediction of OSA risk or facilitating early diagnosis.

**Table 2:** Functional implications of genes linked to snoring and related traits.

**Gene**	**Chromosome**	**Functional Role**	**Associated Traits and Conditions**
*DLEU7*	13	Engaged in the regulation of immune responses and apoptosis.	Related to body mass index, stature, cardiovascular conditions, systolic blood pressure, and deterioration of pulmonary function.
*MSRB3*	12	Encodes a protein that participates in pathways related to protein and lipid metabolism.	Associated with hippocampal volume, lung function, Alzheimer's disease, brain injuries, novelty-seeking personality traits, deafness, and height.
*POC5*	15	Contributes to centrosome function and regulates the cell cycle.	Significant expression is observed in various body parts, including the brain, cerebellum, lungs, blood, and esophagus.
*FTO*	16	Associated with fat mass and obesity.	Previously associated with alcohol intake, coffee intake, and body mass index.
*BCL11B*	14	Involved in neural development and function.	Associated with smoking and other behavioral traits.
*ROBO2*	3	Plays a role in neural development and axon guidance.	Linked with chronotype and musculoskeletal traits such as height and heel bone mineral density.
*SNAP91*	12	Involved in synaptic vesicle trafficking.	Associated with musculoskeletal traits and other behavioral traits.
*BCL2*	18	Regulates cell death and survival.	Associated with musculoskeletal traits and other behavioral traits.
*PRIM1*	17	Encodes a primase involved in DNA replication.	Associated with sleep apnea.
*KDM2B*	19	Involved in epigenetic regulation.	Associated with sleep apnea.
*BDNF*	11	Plays a role in neuronal survival and plasticity.	Associated with sleep apnea.
*STK33*	11	Encodes a serine/threonine kinase involved in cell signaling.	Associated with sleep apnea.
*ANKRD31*	5	Involved in transcriptional regulation.	Associated with sleep apnea.

**Table 3:** Important gene-environment interaction studies related to snoring.

**Study**	**Sample Size (N)**	**Methodology**	**Ethnic Background**	**Population**	**95% Confidence Interval**	**Significant Findings**
Frontiers (2024) [102]	2500	Bidirectional Mendelian randomization	80% European, 10% East Asian, 10% Mixed	18,340 individuals	[1.25, 1.68]	The composition of intestinal bacteria impacts the intensity of snoring.
Sleep Med Res (2024) [103]	1200	Twin and familial studies, GWAS	60% East Asian, 25% Southeast Asian, 15% Other	Various cohorts	[0.10, 0.54]	A genetic tendency for snoring is associated with the DLEU7 and MSRB3 genes
Nature (2023) [104]	3800	Polygenic risk score analysis	50% European, 30% African, 20% Hispanic	Korean cohort (KoGES)	[1.15, 1.42]	Snoring is influenced independently by genetic risk as well as by BMI, age, and lifestyle.

## LIST OF ABBREVIATIONS

*COL1A1*: Collagen, type I, alpha 1 Chain
*COL5A1*: Collagen Type V Alpha 1 Chain
*CRISPR*: Clustered Regularly Interspaced Short Palindromic Repeats
*FGFR2*: Fibroblast Growth Factor Receptor 2
GWAS: Genome-Wide Association Study
*MEIS1*: MeisHomeobox 1
PRISMA: Preferred Reporting Items for Systematic Reviews and Meta-Analyses
RLS: Restless Legs Syndrome
